# Connectivity of the olfactory tubercle: inputs, outputs, and their plasticity

**DOI:** 10.3389/fncir.2024.1423505

**Published:** 2024-05-22

**Authors:** Masahiro Yamaguchi

**Affiliations:** Department of Physiology, Kochi Medical School, Kochi University, Kochi, Japan

**Keywords:** olfactory tubercle, olfactory learning, plasticity, synaptic input, synaptic output

## Abstract

The olfactory tubercle (OT) is a unique part of the olfactory cortex of the mammal brain in that it is also a component of the ventral striatum. It is crucially involved in motivational behaviors, particularly in adaptive olfactory learning. This review introduces the basic properties of the OT, its synaptic connectivity with other brain areas, and the plasticity of the connectivity associated with learning behavior. The adaptive properties of olfactory behavior are discussed further based on the characteristics of OT neuronal circuits.

## Introduction

The olfactory cortex (OC) can be subdivided into areas with distinct structural and functional properties (Neville and Haberly, 2004). The olfactory tubercle (OT) is a component of both the OC and the ventral striatum with medium spiny neurons as its principal neurons; it receives massive dopamine signals from the midbrain (Millhouse and Heimer, 1984; Ikemoto, 2007; Wesson and Wilson, 2011; Mori, 2014). Accordingly, it is involved in odor-guided motivated behaviors, particularly in adaptive learning (Ikemoto, 2007; Wesson and Wilson, 2011; Wesson, 2020). Its input and output connectivity suggests that it lies downstream of the olfactory input–behavioral output pathway, integrates information from various brain regions, and sends outputs to areas related to motivated behaviors (Haberly and Price, 1978). Previously, we showed that the OT has distinct functional domains that represent learned odor-induced attractive and aversive motivated behaviors (Murata et al., 2015). We here introduce our recent study that synaptic inputs to the OT exhibit domain-specific structural plasticity induced by olfactory learning (Sha et al., 2023). Along with this, several characteristics of the neural circuits of the OT and their plasticity are discussed to increase our understanding of the neural mechanisms of olfactory learning.

## The olfactory tubercle as a regulator of motivated olfactory behaviors

**FIGURE 1:**
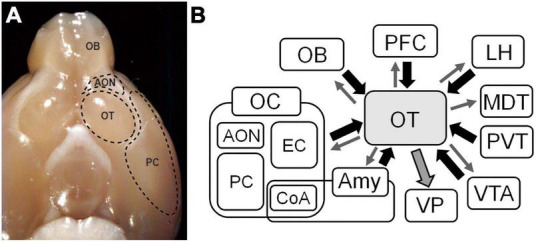
Inputs and outputs of the olfactory tubercle (OT). **(A)** Ventral view of the brain of a mouse. The OT locates posterior to the anterior olfactory nucleus (AON) and medial to the piriform cortex (PC). **(B)** Inputs and outputs of the OT. The OT receives synaptic inputs from various brain areas, and sends outputs reciprocally to those brain areas and massively to the ventral pallidum (VP). OB, olfactory bulb; OC, olfactory cortex; EC, entorhinal cortex; CoA, cortical amygdala; Amy, amygdala; PFC, prefrontal cortex; LH, lateral hypothalamus; MDT, mediodorsal thalamus; PVT, paraventricular thalamus; VTA, ventral tegmental area.

The OC receives direct synaptic inputs from neurons projecting from the olfactory bulb (OB) (Neville and Haberly, 2004) and consists of several distinct areas. In the ventral view of the rodent brain, the OT is readily identified as a round bulge posterior to the olfactory peduncle and medial to the piriform cortex (PC) (Figure 1). As a component of the OC, the OT has a three-layered structure in which axons of OB neurons project to the most superficial layer (layer I). It has properties that are distinct from those of other areas of the OC. While most principal neurons in the OC are glutamatergic pyramidal cells, those of the OT are GABAergic medium spiny neurons (Millhouse and Heimer, 1984). The OT receives massive dopaminergic inputs from the ventral tegmental area (VTA) in the midbrain (de Olmos and Heimer, 1999; Ikemoto, 2007). These properties indicate that the OT is also a component of the striatum, constituting ventral striatum with the nucleus accumbens (NAc). OT neurons express large amounts of acetylcholine esterase (AchE), a characteristic shared with striatal neurons of the NAc and dorsal striatum (Butcher et al., 1975). In the rodent brain, the OT strongly stains with AchE and has a clear boundary separating it from the PC laterally and the diagonal band medially (Paxinos and Franklin, 2019).

As a component of the ventral striatum, it is crucially involved in motivated behaviors (Millhouse and Heimer, 1984; Ikemoto, 2007; Wesson and Wilson, 2011; Mori, 2014). Electrical self-stimulation of the OT is rewarding in rats (Prado-Alcalá and Wise, 1984; Fitzgerald et al., 2014). The OT is a hotspot for cocaine self-administration (Ikemoto, 2003). In addition, as a component of both the OC and ventral striatum, it is involved in odor-motivated behaviors. Innate odor preference is altered by electrical stimulation of the OT (Fitzgerald et al., 2014). In one study, preference for opposite-sex urinary odors was disrupted by suppression of OT activity (DiBenedictis et al., 2015). Activation of the dopaminergic pathway from the VTA to the medial part of the OT reinforces odor preference (Zhang et al., 2017a).

Further, many studies have revealed adaptive properties of the OT. For example, odor-reward association learning potentiates the firing of OT neurons in response to a rewarded odor (Gadziola et al., 2015, 2020; Millman and Murthy, 2020). In one study, following odor-reward or odor-punishment training, neuronal activity in the OT was enhanced in a learning-dependent manner in response to the learned odor (Murata et al., 2015). These observations are consistent with the general notion that the ventral striatum plays crucial roles in the learning, reinforcement, and adaptive modulation of motivated behaviors (Robbins and Everitt, 1996; Averbeck and Costa, 2017). Note that OT-mediated motivated behaviors are not solely odor-guided ones. Therefore, it has been proposed that the OT be called the “tubular striatum” based on its tubular morphology and striatal properties (Wesson, 2020).

## Synaptic connectivity of the OT

The OT belongs to the OC because it receives direct synaptic inputs from OB projection neurons (White, 1965; Scott et al., 1980). However, the overall connectivity of the OT seems different from that of other areas of the OC. While cortical areas have reciprocal connections with other brain areas, the basis of the input to and output from the OT seems that it receives synaptic inputs from various brain areas and sends synaptic outputs to areas linked to motivated behavioral output.

In addition to inputs from the OB, the OT receives intracortical associational inputs from many brain areas (Figure 1B). The PC is the broadest area of the OC and is the source of massive synaptic inputs to the OT (Haberly and Price, 1978; White et al., 2019). Other OC regions project to the OT, including the anterior olfactory nucleus and entorhinal cortex (Haberly and Price, 1978). The prefrontal cortex sends projections to the OT (Berendse and Groenewegen, 1990; Cansler et al., 2023). The amygdala is a complex structure that represent the core of emotions and both cortical and deep amygdaloid nuclei project to the OT (Novejarque et al., 2011). The lateral hypothalamus, which contains various neuropeptide-producing cells, and the paraventricular thalamus (PVT), which mediates motivated behaviors, project to the OT (Groenewegen and Berendse, 1990; Moga et al., 1995). The VTA sends dopaminergic projections to the OT (Ikemoto, 2007).

Contrasting the OT inputs from multiple brain regions, the major output from the OT is considered to the ventral pallidum (VP) (Heimer, 1978) (Figure 1B), a subregion of the ventral basal forebrain complex that regulates emotions, motivation, and motivated behaviors (Soares-Cunha and Heinsbroek, 2023). The VP connects to the reticular formation and extrapyramidal motor systems, and is thought to be a key regulator of motivational behavioral output (Mogenson and Yang, 1991). It is also thought to regulate motivated behaviors via its projections to the lateral hypothalamus and mediodorsal thalamic nuclei (Leung and Balleine, 2015; Faget et al., 2018). Therefore, the OT–VP pathway is considered the central route through which the OT contributes to motivated behaviors.

Many recent studies, including those employing transsynaptic tracing, have revealed various projection targets of the OT, including the PC and anterior olfactory nucleus in the OC (Zhang et al., 2017b), mediodorsal thalamus (Siegel et al., 1977; Price and Slotnick, 1983), posterolateral part of the hypothalamus (Scott and Chafin, 1975), and VTA (Zhang et al., 2017b). Thus, rich reciprocal connections between the OT and other brain areas actually exist. Nonetheless, the prominent property of the OT appears to be its massive output to the VP. This fits with the notion that the OT plays crucial roles in motivational behavior and lies downstream of the sensory input–behavioral output pathway, thereby gathering information from various brain regions and sending output to the VP.

## Plasticity of the synaptic connections of the OT underlying learning-dependent activation of specific OT domains

Previously, we showed that the OT has functional domains that are activated following odor-guided learning. In principle, neutral odors that do not elicit motivated behaviors do not significantly activate the OT. When mice are trained to associate a neutral odor with a food reward and thus become attracted to that odor, the odor stimulus activates the anteromedial domain of the OT (amOT). By contrast, when trained to associate the same odor with electrical shocks to the foot, they become averse to the odor, which activates the lateral domain of the OT (lOT) (Murata et al., 2015). In agreement with this pattern, involvement of the medial part of the OT in odor-attractive behaviors has been reported (DiBenedictis et al., 2015; Zhang et al., 2017a).

The learning-dependent activation of specific OT domains raises questions regarding the underlying plastic mechanisms. Because activation of a brain area depends on synaptic inputs from other brain areas, synaptic inputs to a given OT domain may be potentiated during olfactory-motivated behavior learning, and this may induce domain-specific activation. As a first step to address this possibility, we used optogenetics to activate specific inputs to the OT and examined their plasticity (Sha et al., 2023).

**FIGURE 2:**
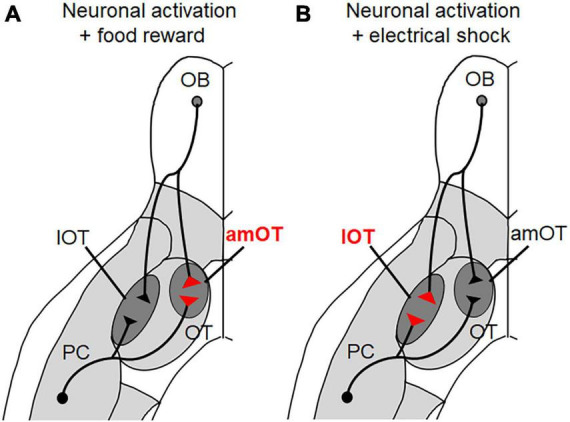
OT domain-specific plasticity of axonal boutons induced by olfactory learning. **(A)** When photoactivation of PC pyramidal neurons and OB projection neurons was associated with a food reward, the axonal boutons terminating in the anteromedial (am) OT domain increased in size. **(B)** When photoactivation of PC pyramidal neurons and OB projection neurons was associated with electrical shock, the axonal boutons terminating in the lateral (l) OT domain increased in size.

Of various synaptic inputs to the OT, inputs from the OB were chosen as being representative of peripheral sensory inputs while inputs from the PC were chosen as being representative of intracortical association inputs. These neurons were modified to express channelrhodopsin-2 fused with fluorescent mCherry protein to enable their photoactivation and morphological analysis (Figure 2). We analyzed the size of the axonal boutons in the photoactivated neurons that terminated in the OT domains, as their size generally correlates with synaptic strength (Murthy et al., 2001; Sammons et al., 2018).

Photoactivation of pyramidal cells in the PC alone did not induce specific behavior in mice. In this case, there were no differences in the sizes of axonal boutons terminating in the amOT and lOT. When photoactivation was associated with a food reward, however, the mice showed food-searching behavior in response to photoactivation. In these mice, the boutons that terminated in the amOT, but not in the lOT, increased in size (Figure 2). By contrast, when the photoactivation was associated with electrical shock, the mice adopted shock-avoiding behavior in response to photoactivation. In these mice, the boutons that terminated in the lOT, but not in the amOT, increased in size. Similar OT domain-specific size development of axonal boutons was observed in OB projection neurons. These observations indicate that both intracortical inputs from the PC and sensory inputs from the OB have plastic potential to induce structural changes in an OT domain-specific manner.

The structural plasticity of the intracortical synapses in the PC is consistent with their role in learning-dependent control of OT activity (White et al., 2019) and with the general notion that plasticity in cortical networks underlies information storage, learning, and adaptive behavior (Feldman, 2009). The structural plasticity of sensory synapses from the OB to the OT domains is intriguing, because several studies have shown that sensory synapses in the OC appear to be hardwired, particularly in the PC, compared to intracortical association synapses (Kanter and Haberly, 1990; Poo and Isaacson, 2007; Johenning et al., 2009; Bekkers and Suzuki, 2013). Odor information from the external environment can reach the OT via as little as two synaptic steps, from olfactory sensory neurons to OB projection neurons and then to OT neurons. Learning-dependent plasticity of the sensory connections from the OB to the OT domains may represent strong adaptive linkage of odor information to behavioral outputs (Doty, 1986).

Because we used artificial optogenetic stimulation, it remains unclear whether similar plastic changes occur during physiological odor stimulation and learning. Nonetheless, the results demonstrate the highly plastic potential of synaptic inputs to the OT domains, which likely underlie the OT domain-specific activation and the expression of appropriate motivated behaviors in a learning-dependent manner. Note that Figure 2 is just a schematic diagram and it is not yet evident whether individual PC and OB neurons send bifurcated axonal projections to both amOT and lOT, or distinct presynaptic populations innervate these functionally distinct OT domains. It remains to be determined whether the plastic change is regulated in individual axonal boutons or in individual neurons. This knowledge may help understand how odor valence is encoded and plastically modulated in the olfactory neuronal circuitry.

## Perspectives on the plasticity of synaptic connections in the OT

Regarding the functional plasticity of the OT synapses, odor-evoked firing activity of OT neurons in awake mice is modulated by the activation of PC to OT inputs (White et al., 2019). In addition to the glutamatergic synaptic inputs, neuromodulators play crucial roles in synaptic plasticity. Dopamine is central to OT-mediated odor learning (Zhang et al., 2017a). Excitatory postsynaptic potentials induced by the lateral olfactory tract stimulation are potentiated by the simultaneous dopamine release (Wieland et al., 2015), and phasic dopamine increases the intensity of excitatory stimulus responses of OT neurons (Oettl et al., 2020). Because dopaminergic input to the medial and lateral OT appears to have a distinct role in neuronal activity (Bhimani et al., 2022), OT domain-specific dopaminergic input may be involved in OT domain-specific plasticity. The OT receives a variety of neuromodulatory signals other than dopamine (Cansler et al., 2020), and biased neuromodulatory signals among OT domains are suggested (Nogi et al., 2020). Given that neuromodulators convey information on various states of the brain and body, the neural mechanisms governing the reception of such information and its integration with synaptic plasticity in the OT during olfactory behavior learning are key to understanding the highly adaptive properties of the OT.

The OT contains D1- and D2-type dopamine receptor-expressing neurons, which have distinct functions in the processing of odor information. Odor-attractive behavior is accompanied by the activation of D1 cells in the amOT, while odor-aversive behavior is accompanied by the activation of D1 cells in the lOT (Murata et al., 2015). Conversely, the activation of D2 cells in the amOT induces aversive behavior (Murata et al., 2019). The differential synaptic plasticity of D1 and D2 cells during olfactory learning has been revealed (White et al., 2019; Gadziola et al., 2020; Martiros et al., 2022). Understanding the structural and functional plasticity of synaptic inputs to D1 and D2 cells during olfactory learning would reveal the differential and combinatory roles of D1/D2 cell-mediated neural pathways in the OT.

Compared to synaptic inputs, there is limited knowledge of synaptic outputs from the OT and their plasticity. Regarding output from the NAc to the VP, cocaine-induced synaptic potentiation/depression has been reported (Baimel et al., 2019). Examining the domain specificity, cell type specificity, and plastic properties of the output from the OT to the VP would provide further insight into the plastic control of olfactory-motivated behaviors. Understanding the function of the OT in the neural network involving cortical and subcortical brain areas and neuromodulatory signaling systems would reveal the neural mechanisms of the adaptive control of odor-guided behaviors.

Lastly, contribution of the OT to innate olfactory behaviors would be worth pursuing. Influence of the OT activity on the preference for conspecific urinary odors (DiBenedictis et al., 2015) suggests OT’s involvement in innate behaviors. In the central amygdala, the same neuronal population controls innate and learned odor-fear behaviors in opposing directions (Isosaka et al., 2015). Comparing the regulatory mechanisms of innate and learned olfactory behaviors in the OT would facilitate the understanding of how these two types of behaviors are related and differentiated in the mammalian brain.

## Author contributions

MY: Writing – review and editing, Writing – original draft, Conceptualization.
